# Integrated Production of Biodiesel and Concentration of Polyunsaturated Fatty Acid in Glycerides Through Effective Enzymatic Catalysis

**DOI:** 10.3389/fbioe.2019.00393

**Published:** 2019-12-20

**Authors:** Gaojian Ma, Lingmei Dai, Dehua Liu, Wei Du

**Affiliations:** ^1^Department of Chemical Engineering, Tsinghua University, Beijing, China; ^2^Key Laboratory of Industrial Biocatalysis, Ministry of Education, Tsinghua University, Beijing, China; ^3^Tsinghua Innovation Center in Dongguan, Dongguan, China

**Keywords:** biodiesel, docosahexaenoic acid (DHA), eicosapentaenoic acid (EPA), lipase, transesterification

## Abstract

DHA (docosahexaenoic acid) and EPA (eicosapentaenoic acid) contained in glycerides have been reported to be more advantageous for their intake than their counterpart in the form of free fatty acid or fatty acid esters. This work attempts to achieve the flexible concentration of DHA and EPA in glycerides as well as biodiesel production via a two-step process catalyzed by lipases. In the first step, several commercial lipases were investigated and Novozym ET2.0 demonstrated the highest potential in selective concentration of DHA and EPA. Over 85% of EPA and other fatty acids were converted to its corresponding FAEEs (fatty acid ethyl esters), while over 80% of DHA remained in glycerides under the optimized conditions. After the first step ethanolysis, the oil phase was subject to molecular distillation and a 97.5% biodiesel (FAEE) content could be obtained. Further flexible enrichment of DHA and EPA in glycerides was realized by immobilized lipase Novozym 435-mediated transesterification of glycerides (remaining in the heavy phase after molecular distillation) with DHA- or EPA-rich EE, and glycerides with 67.1% DHA and 13.1% EPA, or glycerides with 41.1% EPA and 38.0% DHA could be obtained flexibly. This work demonstrated an effective approach for DHA and EPA enrichment combined with biodiesel production through enzymatic catalysis.

## Introduction

Due to the important physiological effects on human body, ω-3 polyunsaturated fatty acids (ω-3 PUFAs), especially eicosapentaenoic acid (EPA, C20:5) and docosahexaenoic acid (DHA, C22:6), have drawn a lot of attention (Hixson et al., 2015). DHA has been recognized to be the most important and abundant ω-3 fatty acid (FA), which is essential in the development of the central nervous systems such as the brain and retina of human body at the early stage of life, indicating the necessity of DHA for infants and pregnant women; meanwhile, EPA has been verified to be effective in preventing cardiovascular diseases in adults (Dyall, 2015). Herein, products enriched in either or both DHA and EPA would be significant for promoting different health benefits for consumers with varying ages, health conditions, or geographic locations (Antypa et al., 2009). Fish oil has been known to be the most important raw material for the enrichment of DHA and EPA. However, the content of DHA and EPA in natural fish oil is limited (Moreno-Perez et al., 2015); thus, the concentration of DHA and EPA from fish oil is necessary and has been broadly researched in recent years (Shimada et al., 1997b; Akanbi et al., 2013; Shanmugam and Donaldson, 2015; Yan et al., 2018; Zhang et al., 2018).

Both chemical and enzymatic methods have been applied in the concentration of DHA and EPA, and enzymatic processes are more recommended considering the milder reaction conditions, higher catalytic activity and substrate selectivity (Akanbi et al., 2013; Shanmugam and Donaldson, 2015). Especially, the enzymatic process has been thought to be more advantageous for maintaining the structure of DHA and EPA, as no aggressive agents are used and the reaction temperature is relatively low during the enzymatic catalysis (Valverde et al., 2012). The lipase's specifics, the reaction process, as well as the acyl donor and the acyl acceptor may influence the enzymatic process, resulting in varied effects on DHA/EPA's enrichment (Shimada et al., 1997b; Valverde et al., 2012, 2013, 2014; Wang et al., 2012; Akanbi et al., 2013; Bhandari et al., 2013, 2015; Shanmugam and Donaldson, 2015; Sampath et al., 2018; Yan et al., 2018; Zhang et al., 2018).

Currently, most of the commercial products of PUFAs are ethyl ester derivatives (Zhang et al., 2018), and lots of researches have been focusing on the enrichment of DHA and EPA in the form of ethyl esters (Shimada et al., 1997b; Yan et al., 2018). However, PUFAs in the form of ethyl esters have lower bioavailability and are harder to be metabolized by human body compared to the form of free fatty acids (FFAs) and glycerides; meanwhile, FFAs are easily oxidized and unstable, thus making glycerides the most suitable form for the supplement of PUFAs in the human body (Wang et al., 2012). There are some researches attempting to obtain natural glyceride concentrates of PUFAs from fish oil by utilizing the selectivity of lipases, including the lipase-mediated selective hydrolysis, alcoholysis, and esterification process. Sampath et al. (2018) concentrated PUFAs in glycerides via selective hydrolysis of Sardine oil catalyzed by a bioimprinted cross-linked *Candida rugosa* lipase, which was effective in catalyzing the hydrolysis of ester linkages of non-PUFA glycerides. Bhandari et al. (2013, 2015) synthesized glyceride mixture enriched in DHA through the immobilized *Candida antarctica* lipase B (CAL-B)-catalyzed esterification of glycerol with DHA-rich FAs obtained by lipase-catalyzed selective esterification of tuna-FFA. Akanbi et al. (2013) enriched DHA in the rest glycerides by lipase-catalyzed ethanolysis of Sardine oil, and <5% of DHA was converted. Valverde et al. (2012, 2013, 2014) realized the selective concentration of DHA and EPA in glycerides via different lipase-mediated alcoholysis of fish oil with different acyl acceptors. At present, although the enrichment of PUFAs in glycerides catalyzed by lipase could be realized by a one-step process like selective hydrolysis or alcoholysis (Valverde et al., 2012, 2013, 2014; Akanbi et al., 2013; Sampath et al., 2018) and a two-step process like selective esterification-esterification (Bhandari et al., 2013, 2015), the related researches paid no attention to the different physiological effects of DHA and EPA, and the ratio of DHA/EPA in the glycerides is settled and non-adjustable; in addition, few researches focused on the utilization of the non-PUFAs in the fish oil, causing the waste of the raw material.

**Scheme 1:**
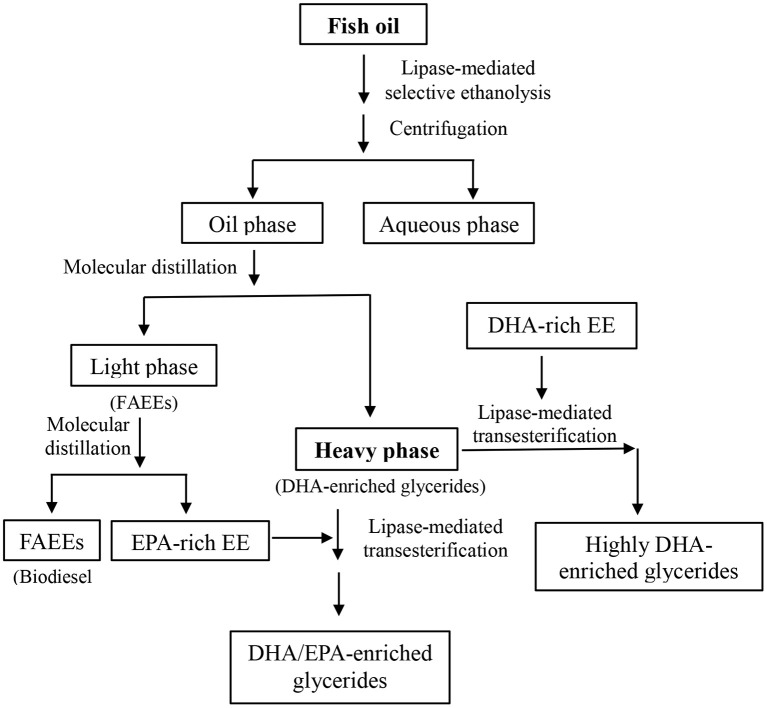
Flexible concentration of DHA and EPA in glycerides and biodiesel preparation from fish oil.

In this work, a lipase-mediated two-step process was proposed to prepare glycerides enriched with flexible ratio of DHA and EPA as well as the preparation of biodiesel simultaneously (Scheme 1).

Several commercial lipases and different acyl acceptors were tested for the investigation of the catalytic selectivity of lipases toward DHA and EPA. Lipase Novozym ET2.0-mediated ethanolysis of fish oil was eventually selected as the first step; after the lipase-mediated first step, molecular distillation was applied to obtain DHA-enriched glycerides and biodiesel (FAEEs). In the second step, immobilized lipase Novozym 435 was adopted to catalyze the transesterification of glycerides (remaining in the heavy phase after the molecular distillation) with DHA- or EPA-rich EE. Through this enzymatic two-step process, flexible enrichment of DHA and EPA in glycerides as well as the preparation of biodiesel from fish oil could be effectively realized, realizing the thorough utilization of fish oil raw material.

## Materials and Methods

### Materials

Fish oil was kindly donated by Hai Zhiyuan Co., Ltd (Guangzhou, China). DHA-rich ethyl esters were purchased from Xi'an Renbang Biological Technology Co., Ltd (Shanxi, China) (76.4% DHA, 13.9% EPA). EPA-rich ethyl esters were purchased from Jiahuitai Biotechnology Development Co., Ltd (Sichuan, China) (48.5% EPA, 31.8% DHA).

Free lipase Novozym ET2.0 (Eversa®Transform2.0) (from *Aspergillus oryzae*, activity ≥100 LCLU/g) was purchased from Novo Industries (Copenhagen, Denmark). Immobilized lipases Novozym 435 (from *Candida antarctica*, activity 10,000 PLU/g) and Lipozyme TL IM (from *Thermomyces lanuginosus*, activity 170 IUN/g) were obtained from Novozymes (Copenhagen Denmark). Porcine Pancreas Lipase (activity 30,000 U/g, PPL) was purchased from Shanghai Yuanye Bio-Technology Co., Ltd. (Shanghai, China).

FA methyl esters (C14:0~C22:6), heptadecanoic acid methyl ester as internal GC standard, and MAGs, DAGs, and TAGs for HPLC analysis were purchased from Sigma-Aldrich (St. Louis, MO). Other reagents of analytical grade were obtained commercially with analytical grade.

### Lipase-Mediated Alcoholysis of Fish Oil for Biodiesel and PUFA Concentration

#### Different Lipase-Catalyzed Methanolysis of Fish Oil for Biodiesel Production

The reactions were conducted as follows: 50.0 g of fish oil, methanol/oil molar ratio 5:1 (equally added four times at 1-h intervals from 0 to 3 h), 2% lipase (w/w, oil), and 10% water (w/w, oil); the mixture was placed in a 250-ml three-neck round bottom flask equipped with a mechanical stirrer at 1,000 rpm and immersed in a thermostat water bath of 45°C for lipase Novozym ET2.0 and PPL. For immobilized lipases Novozym 435 and Lipozyme TL IM, the mixture consisted of 20 g of fish oil, methanol/oil molar ratio 5:1 (equally added four times at 1-h intervals from 0 to 3 h), and 2% lipase (w/w, oil), and placed in a 50-ml conical flask placed in a thermostatic shaking table under 45°C, 180 rpm. Samples were taken from the reaction mixture at specified times and then centrifuged at 80°C, vacuum degree 0.02 MPa to steam the methanol and get the oil layer for analysis.

#### Alcoholysis of Fish Oil Catalyzed by Novozym ET2.0

Alcoholysis of fish oil catalyzed by lipase Novozym ET2.0 was carried out in the same reaction unit described in Different Lipase-Catalyzed Methanolysis of Fish Oil for Biodiesel Production involving Novozym ET2.0. Different reaction conditions were systematically investigated and optimized.

Different acyl acceptors including methanol, ethanol, 1-propanol, and 1-butanol were studied (under the conditions of alcohol adding strategy 1, alcohol/oil molar ratio 5:1, water dosage 10 wt%, lipase dosage 2 wt%, 45°C, and 1,000 rpm).

Alcohol adding strategies (strategy 1: equally added four times at 1-h intervals from 0 to 3 h, strategy 2: equally added eight times at 1-h intervals from 0 to 7 h) were investigated under conditions of ethanol/oil molar ratio 5:1, water dosage 10 wt%, lipase dosage 2 wt%, 45°C, and 1,000 rpm. Water dosage (5, 10, 15, 20%, w/w, oil) was investigated under the conditions of ethanol adding strategy 1, ethanol/oil molar ratio 5:1, lipase dosage 2 wt%, 45°C, and 1,000 rpm. Different alcohol/oil molar ratios (3:1, 4:1, 5:1, and 6:1) were studied under the conditions of ethanol adding strategy 1, water dosage 15 wt%, lipase dosage 2 wt%, 45°C, and 1,000 rpm. Different reaction temperatures (35, 40, 45, and 50°C) were compared under the conditions of ethanol adding strategy 1, ethanol/oil molar ratio 5:1, water dosage 15 wt%, lipase dosage 2 wt%, and 1,000 rpm.

Samples were taken from the reaction mixture at specified times and then centrifuged at 80°C, vacuum degree 0.02 MPa to steam the alcohol and get the oil layer for analysis.

### Molecular Distillation

After Novozym ET2.0-mediated ethanolysis of fish oil, molecular distillation was used to separate fatty acid ethyl esters (FAEEs) from the reaction mixture. The conditions were as follows: 100.0 g crude product mixture, feeding rate 1.0 ml/min, evaporator vacuum 2–5 Pa, rotation speed 200 rpm, and evaporator temperature 110°C. Both the light phase and the heavy phase were collected for properties determination, and then molecular distillation was further used for the separation of EPA-EE from the light phase, with the condition of feeding rate 1.0 ml/min, evaporator vacuum 2–5 Pa, rotation speed 200 rpm, and evaporator temperature 80°C.

### Novozym 435-Catalyzed Transesterification of Glycerides (Remaining in the Heavy Phase After the Molecular Distillation) With DHA- or EPA-Rich EE

The immobilized lipase-catalyzed process was conducted in a 50-ml conical flask and placed in a thermostatic shaking table under 45°C at 200 rpm. The reaction mixture consisted of 5.0 g heavy phase, 20.0 g DHA-EE or EPA-EE {the molar ratio of the exposed hydroxyl group in heavy phase [calculated from the mass ratio of MAGs and DAGs in heavy phase (Zhang et al., 2018)] to ethyl ester of 1:5}, 10% lipase (w/w, heavy phase), and 5.0 g of 3-Å molecular sieves. Samples were taken from the reaction mixture at specified times for GC and HPLC analysis.

### Analytical Method

#### Determination of FA Ester Yield and Analysis of FA Composition of Fish Oil

The calculation of FA ester yield is as follows:

$$\text{FA ester yield }(\%)=\frac{\text{FA ester content}}{\text{the convertible FA content}}\times \text{100}\%$$

where the FA ester content is determined by the following procedure: weigh 6–8 mg of oil sample exactly and 0.6 ml of heptadecanoic acid methyl ester (internal standard, C17:0) ethanol solution (1.11 mg/ml). The sample was mixed by a shaker and then 0.5 μl was injected for GC analysis.

The convertible FA content is determined by the standard procedure AOAC 991.39 (Association of Analytical Communities) with the detailed procedure described as follows: weigh 25.0 mg original fish oil exactly in a tube with 2.0 mg heptadecanoic acid methyl ester as the internal standard, and then add 1.5 ml NaOH-CH_3_OH solution (0.5 mol/L, heated for 15 min at boiling water bath) and 2.0 ml 14% BF_3_-CH_3_OH solution (w/v) (heated for 30 min at boiling water bath). After cooling down to 30–40°C, add 1 ml of hexane and shake for 30 s, then add 5 ml of saturated NaCl solution and shake it until phase separation, take the upper hexane layer out, and inject 0.5 μl of the sample for GC analysis (Lv et al., 2012); at the same time, the FA composition of fish oil could also be determined with the assistance of standards of FA methyl esters.

GC analysis conditions: FID (Flame Ionization Detector) (Agilent 7890A, Agilent Technologies, Santa Clara, CA, USA) and CP-FFAP CB capillary column (25 m × 0.32 mm × 0.30 μm, Agilent J&W GC Columns, Folsom, CA, USA). The initial column temperature was set at 180°C and held for 0.5 min and then heated to 250°C at the rate of 10°C/min and held for 6 min. The temperature of detector and injector was set at 250 and 245°C, respectively.

#### Analysis of the Positional Distribution of FAs in Fish Oil and the FA Composition of Glycerides

The positional distribution of FAs in TAGs of fish oil was determined by the method described in Sahin et al. (2005). In this case, 0.1 g of fish oil, 5 ml of Tris–HCl buffer (pH = 8.0), and 40 mg of PPL were used. The hydrolysis products were applied to a thin-layer chromatography (TLC) plate (50 × 200 mm) coated with silica gel and developed in a TLC tank, the developing solvent was a mixture of hexane/ethyl ether/acetic acid (70:30:1, v/v/v). The bands were sprayed with 0.1% 2,7-dichlorofluorescein in methanol and visualized under ultraviolet (UV) light at 365 nm; then, the corresponding MAGs band was scraped off and further methyl esterified by adding 1 ml of 0.5 mol/L NaOH-CH_3_OH solution (reacting for 15 min in boiling water bath) and 1 ml of BF_3_-CH_3_OH solution (reacting for 30 min in boiling water bath). Then, the extractant with hexane was collected for GC analysis. The GC analysis gave the FA profile at the sn-2 position of original TAGs. From the total content of a given FA in TAGs and the content of this FA at the sn-2 position, the content at the sn-1 or sn-3 position may be calculated by the equation:

$$\begin{array}{l} \text{FA at the sn}1\text{ position }(\%)\\ \;=\frac{3\times \%\text{total FA}-\%\text{FA at the sn}2\text{ position}}{2} \end{array}$$

The analysis of FA composition of glycerides was determined as follows: 50 mg of the oil mixture was applied to the TLC method, then the corresponding glycerides (MAGs, DAGs, and TAGs) bands were scraped off and further methyl esterified, and the extractant with hexane (FA methyl esters) was collected for GC analysis.

#### Analysis of Glycerides by HPLC

The detailed procedure was the same as that described in Ma et al. (2018). The glycerides including triacylglycerides, diacylglycerides, and monoacylglycerides in the oil mixture were analyzed by a Shimadzu 20A HPLC system (Shimadzu Corp., Kyoto, Japan) equipped with an ELAD-LTII low-temperature–evaporative light scattering detector. C18 column (5 μm, 250 mm × 4.6 mm) (Dikma Technology, PLATISIL ODS, China) was used for the separation at 40°C. The mobile phase consisted of acetonitrile–acetic acid (V/V, 99.85:0.15, %) and dichloromethane, which was pumped with a gradient elution program at the rate of 1.5 ml/min (Table 1). The drift pipe temperature was maintained at 40°C, and the nitrogen pressure was controlled at 320 kPa. Fifteen microliters of the sample and 1 ml of hexane were precisely measured and mixed thoroughly, and then 20 μl of the aforementioned mixture was injected for glyceride analysis. The glyceride content was calculated by the standard curve obtained by external standard.

**Table 1:** Gradient elution program of HPLC for separating glycerides.

**Time (min)**	**Acetonitrile–acetic acid (V/V, 99.85:0.15, %)**	**Dichloromethane (V/V, %)**
0	100	0
4	100	0
12	90	10
25	90	10
30	70	30
35	70	30
45	20	80
55	20	80
60	100	0
65	100	0

The glyceride content (monoacylglycerides, diacylglycerides, and triglyceride) was calculated by the standard curve obtained by external standard. The quantitative analysis was accomplished according to the standard lines of MAGs, DAGs, and TAGs established by external method. About 15 μl of the sample and 1 ml of hexane were precisely measured and mixed thoroughly, and then 20 μl of the mixture was injected for glyceride analysis, and the glyceride content was calculated according to the standard curves of MAGs, DAGs, and TAGs.

## Results and Discussion

### Lipase-Mediated Alcoholysis of Fish Oil for Biodiesel Preparation and PUFA Concentration

#### Comparison of Different Lipase-Catalyzed Methanolysis of Fish Oil

It has been reported that different lipases have varied selectivity toward DHA and EPA (Lyberg and Adlercreutz, 2008; Valverde et al., 2012, 2013). In this research, lipases Novozym ET2.0, Novozym 435, PPL, and Lipozyme TL IM were applied in catalyzing the methanolysis of fish oil, and the selectivity of the above lipases toward DHA and EPA was investigated.

**Figure 1:**
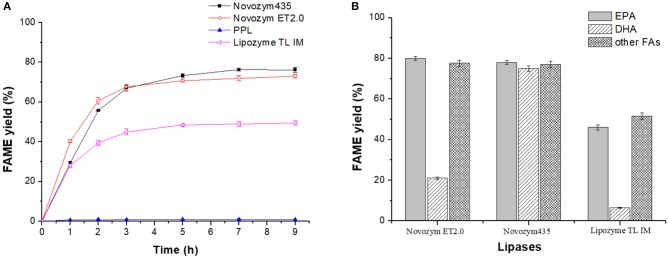
Methanolysis of fish oil catalyzed by lipases. **(A)** Effect of time. **(B)** Effect of lipase.

Figure 1 showed that although Novozym 435 provided the highest FAME yield, the selectivity of the lipase toward DHA and EPA was the weakest. Lipozyme TL IM showed higher selectivity toward DHA but lower FAME yield compared to Novozym 435 and Novozym ET2.0, while PPL showed almost no catalytic activity. Considering the total FAME yield and the selectivity toward DHA and EPA, lipase Novozym ET2.0 had the highest potential during the methanolysis of fish oil compared to other three lipases. To further study whether the different distribution of DHA and EPA in fish oil influenced the catalytic selectivity during lipase-mediated methanolysis, the positional distribution of FAs in original fish oil and the glycerides after the enzymatic methanolysis were investigated, respectively, by the TLC method described in Analysis of the Positional Distribution of FAs in Fish Oil and the FA Composition of Glycerides and the results are shown in Table 2.

**Table 2:** Analysis of positional distribution of FAs.

**FA**	**EPA**	**DHA**	**Other**
**Original fish oil**			
Total (%)	9.5 ± 0.3	16.3 ± 0.6	74.2 ± 1.0
sn-1 (%)	10.1 ± 0.5	15.8 ± 0.6	74.1 ± 0.8
sn-2 (%)	8.3 ± 0.5	17.4 ± 0.5	74.3 ± 0.8
**Residual glycerides mixture after Novozym ET2.0-mediated methanolysis of fish oil**			
Total (%)	7.1 ± 0.2	53.2 ± 0.4	39.7 ± 0.7
sn-1 (%)	7.0 ± 0.5	53.9 ± 0.6	39.1 ± 0.6
sn-2 (%)	7.2 ± 0.3	52.0 ± 0.6	40.8 ± 0.4

**Figure 2:**
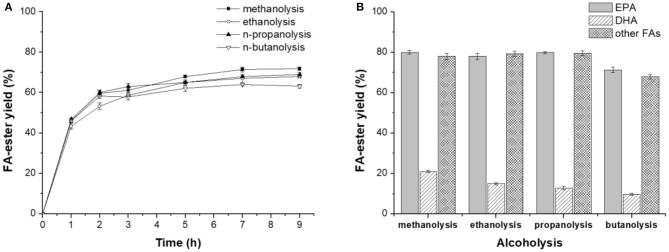
Novozym ET2.0-mediated alcoholysis of fish oil. **(A)** Effect of time. **(B)** Effect of different acyl acceptor.

From Table 2, it could be seen that both DHA and EPA were almost averagely distributed in the three positions of glycerol backbone in original fish oil as well as in the three positions of the glyceride mixture after Novozym ET2.0-mediated methanolysis of fish oil (Mbatia et al., 2010; Tengku-Rozaina and Birch, 2014), indicating that the difference in the conversion of DHA and EPA presented in Figure 2 was due to the varied selectivity of lipase toward them rather than the difference of their original positional distribution.

#### Novozym ET2.0-Mediated Alcoholysis of Fish Oil With Different Acyl Acceptors

The above study revealed that lipase Novozym ET2.0 had good performance in catalyzing the methanolysis of fish oil for the selective concentration of DHA and EPA. Further, Novozym ET2.0-mediated alcoholysis of fish oil with different acyl acceptors (methanol, ethanol, 1-propanol, and 1-butanol) was carried out since different acyl acceptors have been reported to affect the catalytic performance of lipase (Shimada et al., 1997a; Valverde et al., 2014). As shown in Figure 2A, it could be seen that the highest ester yield was achieved with methanol, but the selectivity toward DHA was lower than that of the other three alcohols (Figure 2B). With ethanol and 1-propanol as the acyl acceptors, high ester yield and selectivity could be achieved. The spatial conformation of the binding site of lipase is a tunnel-like cavity (Pleiss et al., 1998), and the binding process of DHA chain may be harder if the steric hindrance of the alcohol is higher, thus reducing the conversion degree of DHA to ester, while the binding process of other FA chains would not be affected when using different alcohols as acyl acceptors (except for 1-butanol).

#### Optimization on Novozym ET2.0-Mediated Selective Ethanolysis of Fish Oil

**Figure 3:**
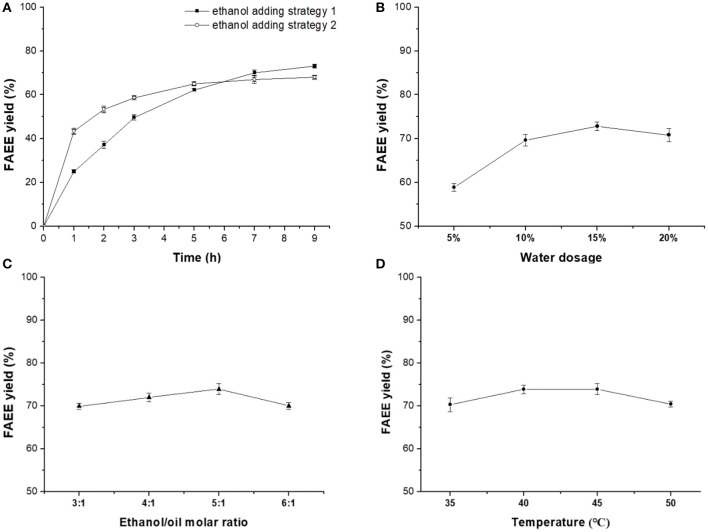
Optimization on Novozym ET2.0-mediated ethanolysis of fish oil. **(A)** Effect of time. **(B)** Effect of water dosage. **(C)** Effect of ethanol/oil molar ratio. **(D)** Effect of temperature.

In order to obtain higher ester yield and promote enzymatic selective catalysis toward DHA and EPA, effect of ethanol adding strategy, water dosage, ethanol/oil molar ratio, and reaction temperature were optimized systematically in the following study. Figure 3A showed that ethanol adding strategy 2 (equally added eight times at 1-h intervals from 0 to 7 h) provided higher FAEE yield than strategy 1 (equally added four times at 1-h intervals from 0 to 3 h). The deactivation of ethanol on lipase could be reduced by increasing the adding times, lowering the amount of ethanol each time.

A certain amount of water has been demonstrated to be necessary for the Novozym ET2.0-mediated process (Lv et al., 2017). In this research, water dosages of 5, 10, 15, and 20% (w/w, oil) were selected for investigating the effect of water on Novozym ET2.0-mediated ethanolysis of fish oil (Figure 3B). Results showed that FAEE yield increased with water dosage from 5 to 15% and decreased when the water dosage was 20%.

The effect of ethanol/oil molar ratio (3:1, 4:1, 5:1, and 6:1) on Novozym ET2.0-mediated ethanolysis of fish oil is shown in Figure 3C. Higher FAEE yield was obtained when the ethanol/oil molar ratio was increased from 3:1 to 5:1, but the FAEE yield decreased when the molar ratio was further increased from 5:1 to 6:1.

Reaction temperatures of 35, 40, 45, and 50°C were selected to investigate the effect of temperature on the ethanolysis process. Figure 3D shows that the FAEE yield increased when the temperature was increased from 35 to 40°C and maintained stable during 40–45°C, and the FAEE yield decreased when the temperature was further enhanced.

**Figure 4:**
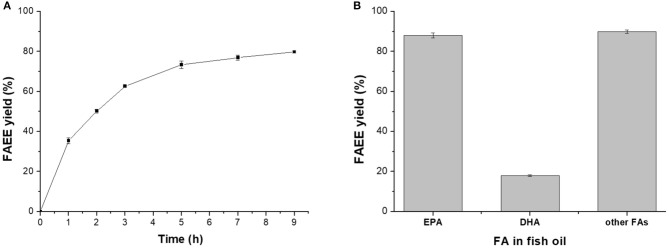
Lipase-mediated ethanolysis of fish oil under optimized conditions. **(A)** Effect of time. **(B)** Conversion of different FAs to their esters.

Under the optimized conditions (ethanol adding strategy 1, water dosage 15 wt%, ethanol/oil molar ratio 5:1, and 40°C), a FAEE yield of 79.8% could be achieved after 9 h of reaction (Figure 4A), where more than 85% of EPA and other FAs were converted to their corresponding esters, while most of the DHA remained in the glycerides (Figure 4B), providing basis for further rational concentration of DHA or/and EPA in triglycerides.

### Separation of Biodiesel (Ethyl Esters) From Glycerides With Molecular Distillation

After the above lipase-mediated ethanolysis, the reaction mixture was subject to centrifugation and the oil phase was collected for further molecular distillation to separate ethyl esters from glycerides (Wang et al., 2009), which would avoid the potential negative effect of high temperature on the stability of DHA.

The FAEE content in heavy phase was decreased to 10.8% after one-stage molecular distillation and the FA composition of heavy phase and light phase are listed in Table 3.

**Table 3:** FA composition of heavy phase and light phase.

**Fatty acid (%)**	**Original fish oil**	**Heavy phase**	**Light phase**
C14:0 (Myristic acid)	6.8 ± 0.1	4.4 ± 0.1	8.9 ± 0.1
C16:0 (Palmitic acid)	20.9 ± 0.6	11.8 ± 0.5	26.5 ± 0.3
C16:1 (Palmitoleic acid, ω-7)	7.4 ± 0.3	5.9 ± 0.2	9.2 ± 0.2
C18:0 (Stearic acid)	4.2 ± 0.2	1.9 ± 0.1	4.4 ± 0.1
C18:1 (Oleic acid, ω-9)	16.6 ± 0.5	9.0 ± 0.3	20.0 ± 0.3
C18:2 (Linoleic acid, ω-6)	3.3 ± 0.2	1.7 ± 0.1	3.7 ± 0.1
C18:3 (Linolenic acid, ω-3/6)	2.0 ± 0.2	0.2 ± 0.0	2.5 ± 0.1
C20:1 (Eicosenoic acid, ω-9)	5.2 ± 0.3	4.9 ± 0.1	5.6 ± 0.1
C20:4 (Eicosatetraenoic acid, ω-3/6)	1.2 ± 0.1	1.1 ± 0.1	1.1 ± 0.1
C20:5 (Eicosapentaenoic acid, ω-3)	9.5 ± 0.3	8.4 ± 0.2	9.6 ± 0.2
C22:1 (Docosenoic acid, ω-9)	6.6 ± 0.2	4.8 ± 0.2	7.0 ± 0.1
C22:6 (Docosahexanenoic acid, ω-3)	16.3 ± 0.5	50.0 ± 0.7	1.5 ± 0.1

After the molecular distillation, the DHA content in heavy phase was promoted to 50%, significantly higher than that of original fish oil (16.3%), and most DHA was in the form of MAGs and DAGs, realizing the first-step concentration of DHA in glycerides effectively, while it can be used as the “bone” for further concentration of DHA and EPA onto the glycerides, as shown in Scheme 1.

The light phase was further subject to the second-stage molecular distillation for the separation of EPA-EE and FAEEs (biodiesel) and a FAEE (biodiesel fraction) content of 97.5% could be obtained.

Through the above lipase-mediated selective ethanolysis and molecular distillation, enrichment of DHA in glycerides, concentration of EPA-EE, and biodiesel preparation were achieved successfully. The DHA-enriched glyceride mixture in heavy phase consisted of 65.8% MAGs and 34.2% DAGs, and in the following study, further concentration of DHA and EPA in glycerides was explored by introducing extra DHA or EPA into the glyceride backbone through lipase-catalyzed transesterification of DHA or EPA enriched EE (ethyl ester) with the glycerides contained in heavy phase.

### Further Concentration of DHA or EPA in Glycerides Through Lipase-Catalyzed Transesterification

To realize the flexible and high concentration of DHA and EPA in glycerides, lipase Novozym 435-catalyzed transesterification of heavy phase (obtained from the first step) with DHA- or EPA-rich EE was carried out.

**Figure 5:**
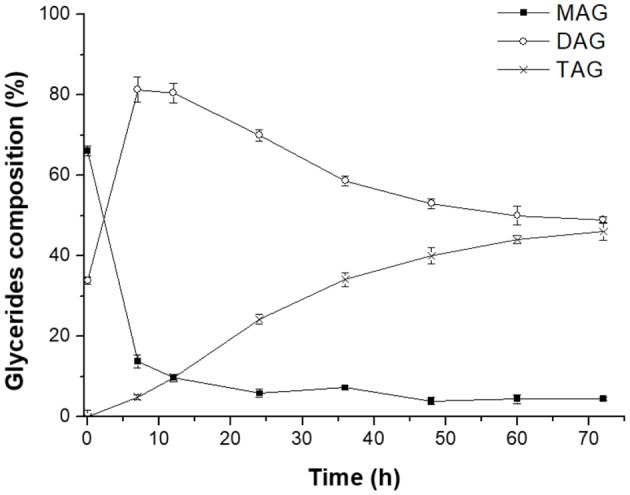
Novozym 435-catalyzed transesterification of heavy phase with DHA-rich EE.

**Scheme 2:**
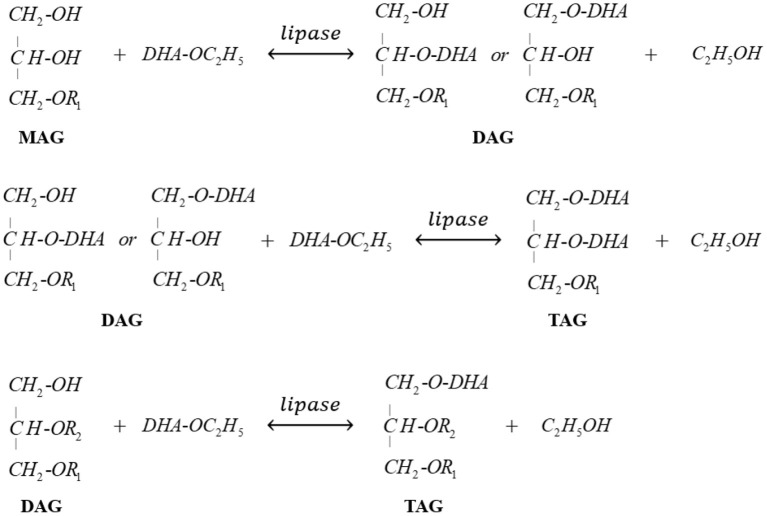
Enzymatic transesterification of glycerides in heavy phase (MAGs and DAGs) with DHA-EE.

Novozym 435-catalyzed transesterification of heavy phase with DHA-rich EE was firstly carried out to further promote the enrichment of DHA in glycerides. Figure 5 clearly indicated that most of MAGs was consumed to produce DAGs at the early stage (0–7 h). Meanwhile, when the reaction time exceeded 12 h, the content of DAGs in glycerides decreased rapidly due to the formation of TAGs. After 72 h reaction, the glyceride mixture consisted of 46.2% TAGs, 49.3% DAGs, and 4.5% MAGs, indicating that most MAGs converted to DAGs or TAGs successfully with the catalysis of Novozym 435. The related reactions involved in this process are presented in Scheme 2.

**Figure 6:**
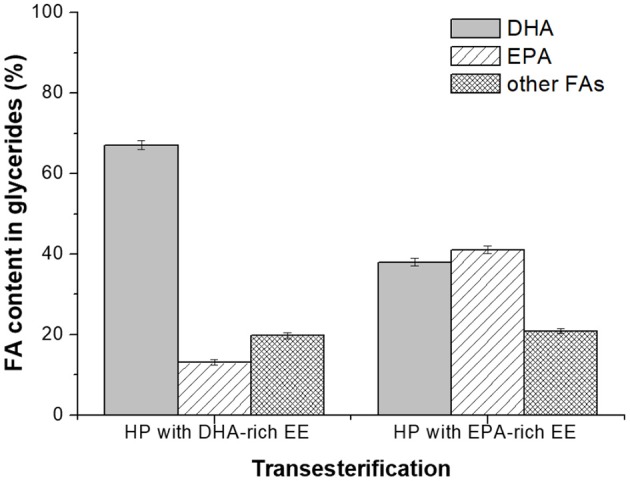
Novozym 435-catalyzed transesterification of glycerides with DHA- or EPA-rich EE (72 h).

After Novozym 435-catalyzed transesterification of heavy phase with DHA-rich EE for 72 h, the DHA content in glycerides was significantly promoted to 67.1% with the total content of PUFAs around 80.2% (Figure 6), which could be used as an ideal healthcare product for infants and pregnant women (Wang et al., 2009; Rogers et al., 2013; Dyall, 2015; Echeverría et al., 2017). For some cases, enrichment of DHA and EPA simultaneously in glyceride is preferred, since it can be used as the product for preventing cardiovascular diseases in adults. Herein, Novozym 435-mediated transesterification of heavy phase with EPA-rich ethyl esters was also investigated as described in Novozym 435-Catalyzed Transesterification of Glycerides (Remaining in the Heavy Phase After the Molecular Distillation) With DHA- or EPA-Rich EE and EPA content in glycerides was promoted to 41.1% with a DHA content of 38.0%. The investigation indicated that through Novoyzm 435-mediated transesterification, either DHA or EPA could be effectively enriched in glycerides, with the total content of PUFAs being more than 75%.

## Conclusion

This work introduced a novel process to achieve the high and flexible concentration of DHA and EPA in glycerides combined with the effective production of biodiesel. Novozym ET2.0 demonstrated high potential in selective enrichment of DHA and EPA for its potent selectivity toward them. Through Novozym ET2.0-mediated selective ethanolysis, most EPA and other FAs were converted to its corresponding FAEE, while most DHA remained in glycerides. The common FAEEs (biodiesel fraction) could be separated effectively from EPA-EE through molecular distillation and the final biodiesel content of 97.5% could be obtained. Further enrichment of DHA and EPA in glycerides could be achieved by Novozym 435-catalyzed transesterification of glyceride (remaining in heavy phase after the molecular distillation) with DHA- or EPA-rich EE and glycerides with 67.1% DHA and 13.1% EPA, or glycerides with 41.1% EPA and 38.0% DHA could be obtained flexibly, providing alternative reasonable nutrition supplement for different consumer groups. This work demonstrated a novel process for the thorough utilization of fish oil, with which the combined production of biodiesel as well as the flexible enrichment of DHA and EPA in glycerides could be achieved effectively, having great prospect for practical industry application.

## Data Availability Statement

All datasets generated for this study are included in the article/supplementary material.

## Author Contributions

GM is mainly responsible for carrying out the experiment and LD is responsible for some instrumental analysis. DL offers constructive suggestions for the research. WD is generally responsible for the experiment design and for coordinating the research.

### Conflict of Interest

The authors declare that the research was conducted in the absence of any commercial or financial relationships that could be construed as a potential conflict of interest.
